# Pathogens of Wild Birds: Prevalence and Molecular and Morphological Characterization

**DOI:** 10.3390/pathogens14080825

**Published:** 2025-08-20

**Authors:** Alazne Díez-Fernández, Rafael Gutiérrez-López

**Affiliations:** 1 Department of Zoology, University of Extremadura, Avda Elvas s/n, 06071 Badajoz, Spain; aldiezf@unex.es; 2 National Center of Microbiology, Health Institute Carlos III, Ctra Pozuelo, 28, 28222 Madrid, Spain; 3 Consorcio de Investigación en Biomedicina en Red de Enfermedades Infecciosas (CIBERINFEC), C/Monforte de Lemos, 3-5, 28029 Madrid, Spain

## 1. Introduction

Wild birds are exposed to multiple infections by pathogenic microorganisms. These microorganisms could play an essential role in the evolution of avian populations, affecting them physiologically, such as in their survival and reproductive success, as well as behaviorally, by modifying their migratory patterns [1,2,3,4]. Parasites that can affect wild bird populations can be classified as ectoparasites (e.g., *ticks*, *mites*, *flies*, etc.) and endoparasites (e.g., protozoa, *filariae*). Furthermore, many wild bird species can play an essential role as reservoirs for several viral pathogens, some of which have considerable zoonotic potential, posing risks to both wildlife and human health (e.g., *West Nile virus*, *avian influenza*) [5]. The current global change we are experiencing, which results in climate change, land-use changes, and the globalization of human activities, has major consequences for the dynamics of avian diseases, leading to an expansion of the distribution of pathogens and changes in host–parasite interactions.

This Special Issue compiles seven studies that improve our understanding of the epidemiology, molecular diversity, and surveillance of parasites and pathogens affecting wild birds. The contributions cover a wide range of topics, from infections by ectoparasites and haemosporidian parasites to viral infections such as West Nile virus and avian influenza. In addition, the importance of surveillance programs for monitoring avian-borne zoonotic diseases is emphasized, particularly those associated with tick-borne pathogens, which have increased in recent years. Thanks to these contributions, highly valuable information has been obtained about the complex network of interactions between birds, their parasites, and the ecology and epidemiology of avian diseases.

## 2. Ectoparasites: External Threats to Wild Birds

Obligate ectoparasites, such as lice, ticks, and louse-flies, among others, play an important role in the health of wild birds. These parasites feed on the blood, feathers, or skin of their hosts, which can cause direct harm by reducing their physical condition, potentially affecting their thermoregulation, and increasing their vulnerability to predators [6]. Moreover, many of these ectoparasites can act as vectors for various endoparasites and pathogens, facilitating the transmission of bacterial, protozoan, and viral infections. Therefore, understanding the distribution and prevalence of ectoparasites in bird populations is essential for assessing their ecological impact and developing bird conservation strategies. However, the accurate identification of ectoparasites is sometimes difficult due to a lack of specific keys and expert entomologists. The use of molecular analysis has provided valuable insights into the taxonomy of ectoparasite groups [7]. Regarding mites, the family Rhinonyssidae also includes hematophagous endoparasite mites that parasitize birds. However, they have traditionally been neglected despite their potential role as vectors of various pathogens [8]. Traditionally, the taxonomy of the group has been based on morphometric characteristics, which makes it difficult to identify in many closely related species groups. In this Special Issue, Sánchez-Carrion et al. [9] performed an assessment of the sequence variation in the D1–D3 domains of the 28S rRNA gene among different genera and species of rhinonyssidae mites, evaluating its suitability for phylogenetic reconstruction and taxonomic resolution within the group. This study offers valuable information on the high-resolution potential of the 28S rRNA gene. The results indicate that 28S is a suitable marker. This study is consistent with previous studies that found limited intraspecific variation in avian intranasal mites using markers such as ITS1-ITS2 and/or COI. The results of this study indicate that the 28S rRNA fragment is effective in addressing several taxonomic and phylogenetic questions at the genus and species levels; however, additional molecular markers should be explored to resolve intergeneric relationships and distinguish closely related species within species complexes. Furthermore, the authors highlight the need to review the classification of this family, noting that specific morphometric and molecular identification would be essential to determine the discriminatory characteristics of this group of mites.

## 3. Endoparasites: Internal Infections and Their Impact on Avian Health

Although ectoparasites are observable on the body surface, endoparasites often represent a more covert threat by targeting the internal organs and tissues. Among the most thoroughly investigated avian endoparasites are the haemosporidian protozoa, encompassing genera such as *Plasmodium*, *Haemoproteus*, and *Leucocytozoon*. These microorganisms are transmitted by hematophagous arthropods and are responsible for avian malaria and other blood-related pathologies, which may lead to significant physiological stress and, in some cases, host mortality [10]. Investigating haemosporidian parasites is crucial not only for understanding their impact on wild bird populations but also for gaining insight into host–parasite coevolution and interactions [11].

Within haemosporidians, *Haemoproteus* species (apicomplexa: *Haemosporida*) are among the most widespread avian parasites, exhibiting remarkable genetic diversity—with over 1900 identified mitochondrial lineages—and comprising at least 178 described morphospecies. Despite their diversity, the lifecycles of many *Haemoproteus* species remain poorly elucidated. One species of particular interest is *Haemoproteus majoris*, associated with several lineages, including hCCF5, hPARUS1, hPHSIB1, hPHYBOR04, and hWW2, reported in 54 avian species across Asia, Europe, Africa, and North America [12]. The hCWT4 lineage, tentatively linked to *H. majoris* [13], had not been formally described based on morphological features. In this Special Issue, Duc et al. [14] addressed this gap by characterizing the hCWT4 lineage morphologically as *H. majoris* and conducting a comparative analysis of exoerythrocytic stages across different lineages (hCCF5, hCWT4, hPARUS1, hPHSIB1, and hWW2) in multiple avian hosts and seasons. Their findings revealed that infections associated with the hCWT4 lineage consistently exhibited only megalomeronts. Across the 14 avian species examined, the exoerythrocytic stages were generally consistent, especially among lineages hCCF5, hPARUS1, and hPHSIB1. Interestingly, the megalomeronts of hWW2 and hCWT4 showed greater morphological similarity to each other than to the other lineages.

Within the haemosporidians, *Leucocytozoon* remains one of the least explored, likely due to the morphological complexity and diagnostic challenges associated with their identification [15]. These parasites are also commonly observed at low parasitemia levels and often appear deformed in blood smears. In this Special Issue, Chagas et al. [16] investigated the types of host blood cells targeted by *Leucocytozoon* in naturally infected passerine birds and examined whether this cellular tropism correlates with phylogenetic relationships. Their results showed that *Leucocytozoon* can invade a variety of blood cell types, including erythrocytes, lymphocytes, and thrombocytes. Although the underlying mechanisms driving this cellular specificity remain unknown, the authors suggest that this trait may carry phylogenetic significance and warrants further investigation.

Beyond haemosporidians, this Special Issue also features a novel contribution addressing a different group of avian endoparasites with zoonotic relevance. Solarczyk et al. [17] present the first molecular evidence of *Encephalitozoon cuniculi*, a microsporidian parasite, in migratory waterfowl (*Anser* spp.) in Poland. Although microsporidia are phylogenetically distinct from apicomplexan parasites, their capacity for cross-species transmission and environmental persistence raises significant One Health concerns. This study demonstrates that *E. cuniculi*, commonly associated with infections in mammals including humans, can also be detected in wild migratory birds, reinforcing the role that these avian hosts play as potential reservoirs and disseminators of zoonotic pathogens.

## 4. Viral Pathogens in Wild Birds: Emerging and Zoonotic Threats

In addition to parasites, wild birds are frequently involved in the transmission cycles of diverse viruses. Some of these viruses are highly pathogenic and have the potential to spread across species barriers, raising concerns for both avian and human health. Two of the most significant avian viruses are West Nile virus (WNV) and avian influenza. WNV is a mosquito-borne flavivirus that circulates primarily among birds but can also infect humans and other mammals. Avian influenza viruses, particularly highly pathogenic strains such as H5N1, have been responsible for major outbreaks in both wild and domestic bird populations, sometimes spilling over into human populations with serious consequences.

In this Special Issue, Talmi-Frank et al. [18] tested the hypothesis that high viremia in birds, specifically crows, would lead to greater genetic diversity within the birds’ peripheral blood mononuclear cells. This could partly explain the previously observed host-specific differences in genetic diversity and fitness. To do so, the authors used experimental infections of cells and birds with genetically marked West Nile virus (WNV) to study viral diversity at the cellular level. The results showed that crows harbor a more diverse viral population than robins. Furthermore, crows retain rare virus variants more frequently. This suggests that the higher viral load in crows allows for the persistence of rare variants, possibly due to a phenomenon of viral complementation. The authors also conclude that purifying selection acts less intensely in crows, due to their high susceptibility, high viremia, and presence of multiple viral infections per cell.

Another avian virus on the rise is avian influenza. In recent years, it has spread throughout the world, affecting millions of birds of different species. In this Special Issue, Mihiretu et al. [19], during the winter of 2021–2022, carried out avian influenza virus (AIV) monitoring at a wintering area for migratory birds of the Anatidae family in Japan. They detected a highly pathogenic virus (H5N8) with an unusual genetic combination, in addition to four low-pathogenic viruses. Comprehensive genomic analysis revealed that six of the eight segments of the H5N8 virus (named NK1201) were closely related to strains responsible for outbreaks on poultry farms in Japan in November 2021. However, the PB1 and NP genes came from low-pathogenic viruses (H7N7 and H1N8) identified at the same location and period. This indicates that the NK1201 virus emerged as a result of genetic reassortment between high- and low-pathogenic viruses present in that environment. Furthermore, experiments with chickens showed that NK1201 had a slightly different infectivity than other similar strains, possibly due to genes inherited from low-pathogenic viruses. These findings confirmed the emergence of a new H5N8 genotype, and the authors emphasized the importance of monitoring these wintering sites for emerging variants that may pose a risk to poultry, other livestock, and humans.

## 5. Epidemiological Surveillance: A Key Approach in Avian and Zoonotic Health

One of the most effective tools for managing avian diseases is surveillance. By tracking pathogen prevalence in wild bird populations, researchers can identify trends, anticipate outbreaks, and implement measures to prevent the spread of infections. Surveillance is particularly crucial for zoonotic diseases, as birds often act as reservoirs for pathogens that can spill over into human populations.

In a key study in this Special Issue, Loureiro et al. [20] examine the role that birds play in the transmission of tick-borne zoonotic pathogens. Ticks are vectors for several bacterial and viral infections, including *Borrelia* spp. (the causative agents of Lyme disease) and *Anaplasma* spp., which can affect both wildlife and humans. This study highlights the complex interactions between birds, ticks, and zoonotic pathogens, emphasizing the need for integrative surveillance programs that consider both avian and vector-borne disease dynamics.

## 6. Conclusions

The research compiled in this Special Issue provides a comprehensive overview of the diverse parasites and pathogens affecting wild birds. From the burden of ectoparasites to the hidden threats posed by endoparasitic and emerging viral pathogens, these studies collectively highlight the intricate relationships between birds, their parasites, and their changing environments. Furthermore, the emphasis on surveillance underscores the necessity of proactive monitoring to mitigate the risks associated with avian diseases, particularly those with zoonotic potential.
